# Supplementary material for: Efficacy and safety of cAMP signalling-biased GLP-1 analogue ecnoglutide monotherapy versus placebo in patients with type 2 diabetes (EECOH-1): a multi-centre, randomised, double-blind, placebo-controlled, phase 3 trial
Source: Nat Commun. 2026 Jan 7;17:1420. doi: 10.1038/s41467-025-68165-7 (PMC12881390; doi:10.1038/s41467-025-68165-7)
Supplement: Supplementary file 1 — Supplementary Information [file 41467_2025_68165_MOESM1_ESM.pdf]

Efficacy and safety of cAMP signalling-biased GLP-1 analogue ecnoglutide monotherapy versus placebo in patients with type 2 diabetes (EECOH-1): a multi-centre, randomised, double-blind, placebo-controlled, phase 3 trial

SUPPLEMENATRY INFORMATION

Table of Contents

Full list of ethics committees 2
Dosing regimen 2
Usage and dosage 2
Run-in period 3
Treatment period 3
Dose modification criteria 3
Treatment of missed dose 3
Rescue therapy 3
Complete eligibility criteria 4
Hypothesis testing and multiplicity 5
Supplementary Fig. 1: Time from treatment initiation to discontinuation 7
Supplementary Fig. 2: Cumulative distribution of change from baseline in HbA_{1c} at week 24 8
Supplementary Fig. 3: Sensitivity analysis of estimated treatment differences for change from baseline in HbA_{1c} at week 24 9
Supplementary Fig. 4: Subgroup analysis of estimated treatment difference for change from baseline in HbA_{1c} at week 24 10
Supplementary Fig. 5: Cumulative distribution of change from baseline in bodyweight at week 24 11
Supplementary Fig. 6: Incidences of decreased appetite and diarrhoea over time 12
Supplementary Fig. 7: Changes from baseline in lipase and amylase over time 13
Supplementary Fig. 8: Study design 14
Supplementary Table 1: Additional efficacy measures at week 24 15
Supplementary Table 2: Efficacy measures at week 52 16
Supplementary Table 3: Pulse rate and blood pressure at baseline and week 24 17
Supplementary Table 4: Treatment-emergent adverse events (TEAEs) during the open-label treatment period 18
Supplementary Table 5: Treatment-emergent adverse events (TEAEs) during the entire treatment period 19
Supplementary Note 1: CONSORT Reporting Checklist 20

Full list of ethics committees

Site	Ethics committee
Nanjing Drum Tower Hospital, The Affiliated Hospital of Nanjing University Medical School	Medical Ethics Committee of Drum Tower Hospital Affiliated to Nanjing University School of Medicine
Nanjing First Hospital	Ethics Committee of Nanjing First Hospital
Nanjing Jiangning Hospital	Ethics Committee of Nanjing Jiangning Hospital
The Second Affiliated Hospital of Nanjing Medical University	Medical Ethics Committee of the Second Affiliated Hospital of Nanjing Medical University
The First Affiliated Hospital of Soochow University	Medical Ethics Committee of the First Affiliated Hospital of Soochow University
Central Hospital Affiliated to Shandong First Medical University	Medical Ethics Committee of Jinan Central Hospital
Jining First People's Hospital	Medical Ethics Committee of Jining First People’s Hospital
Yiyang Central Hospital	Clinical Trial Ethics Committee of Yiyang Central Hospital
The First Affiliated Hospital of Nanyang Medical College	Clinical Trial Ethics Committee of the First Affiliated Hospital of Nanyang Medical College
Shiyan People's Hospital	Drug Clinical Trial Ethics Committee of Shiyan People's Hospital
The Second Hospital of Anhui Medical University	Drug Clinical Trial Ethics Committee of the Second Affiliated Hospital of Anhui Medical University
Binzhou Medical University Hospital	Clinical Trial Ethics Committee of Binzhou Medical University Hospital
Shanghai Pudong New Area People's Hospital	Clinical Trial Ethics Committee of Shanghai Pudong New Area People's Hospital
Hebei Petro China Central Hospital	Clinical Trial Ethics Committee of Hebei Petro China Central Hospital
The First Affiliated Hospital of Henan University of Science & Technology	Medical Ethics Committee of the First Affiliated Hospital of Henan University of Science & Technology
The Second Affiliated Hospital of Zhengzhou University	Medical Ethics Committee of the Second Affiliated Hospital of Zhengzhou University
Yueyang People's Hospital	Drug Clinical Trial Ethics Committee of Yueyang People's Hospital
Yueyang Central Hospital	Clinical Trial Ethics Committee of Yueyang Central Hospital
The Third Hospital of Changsha	Ethics Committee of Changsha Third Hospital
Daqing People's Hospital	Ethics Committee of Daqing People's Hospital
The People's Hospital of Liaoning Province	Ethics Committee of Liaoning Provincial People's Hospital
Yibin Second People's Hospital	National Drug Clinical Trial Ethics Committee of Yibin Second People's Hospital
The Affiliated Hospital of Xuzhou Medical University	Medical Ethics Committee of the Affiliated Hospital of Xuzhou Medical University
Beijing Boai Hospital	Ethics Committee of the Drug Clinical Trial Institution of Beijing Boai Hospital
Shijiazhuang People's Hospital	Drug/Medical Device Clinical Trial Ethics Committee of Shijiazhuang People's Hospital
Nanyang Second General Hospital	Clinical Trial Ethics Committee of Nanyang Second People's Hospital
Pingxiang People's Hospital	Ethics Committee of Pingxiang People's Hospital

Dosing regimen

Usage and dosage

The investigational product is administered via subcutaneous injection, once weekly. It is recommended that subjects try to inject on the same day of the week, and may inject at any time of the day, without the need to administer the drug based on meal times.

Run-in period

Inject 0.15 mL vehicle of Ecnoglutide Injection and administer for 4 consecutive weeks.

Treatment period

For subjects randomly assigned to the 1.2 mg Ecnoglutide Injection group:

- Double-blind core treatment period: A starting dose of 0.3 mg of Ecnoglutide Injection administered for 4 weeks; up-titrate to 0.6 mg administered for 4 weeks; up-titrate to 1.2 mg administered for 16 weeks, for a total of 24 weeks of treatment, with unblinding after the last dose.
- Open-label treatment period: Maintenance dose of 1.2 mg for 28 weeks.
- The total dosing period is 52 weeks.

For subjects randomly assigned to the 1.2 mg volume-matching placebo group:

- Double-blind core treatment period: A starting dosing volume of 0.15 mL of Ecnoglutide Injection vehicle administered for 4 weeks; up-titrate to 0.3 mL administered for 4 weeks; up-titrate to 0.6 mL administered for 16 weeks, for a total of 24 weeks of treatment, with unblinding after the last dose.
- Open-label treatment period: After the unblinding, a starting dose of 0.3 mg of Ecnoglutide Injection administered for 4 weeks; up-titrate to 0.6 mg administered for 4 weeks; up-titrate to 1.2 mg administered for 20 weeks, for a total of 28 weeks of treatment.
- The total dosing period is 52 weeks.

For subjects randomly assigned to the 0.6 mg Ecnoglutide Injection group:

- Double-blind core treatment period: A starting dose of 0.3 mg of Ecnoglutide Injection administered for 4 weeks; up-titrate to 0.6 mg administered for 20 weeks, for a total of 24 weeks of treatment, with unblinding after the last dose.
- Open-label treatment period: Maintenance dose of 0.6 mg for 28 weeks.
- The total dosing period is 52 weeks.

For subjects randomly assigned to the 0.6 mg volume-matching placebo group:

- Double-blind core treatment period: A starting dosing volume of 0.15 mL of Ecnoglutide Injection vehicle administered for 4 weeks; up-titrate to 0.3 mL administered for 20 weeks, for a total of 24 weeks of treatment, with unblinding after the last dose.
- Open-label treatment period: After the unblinding, a starting dose of 0.3 mg of Ecnoglutide Injection administered for 4 weeks; up-titrate to 0.6 mg administered for 24 weeks, for a total of 28 weeks of treatment.
- The total dosing period is 52 weeks.

Dose modification criteria

The predominant adverse reaction of GLP-1 analogs is gastrointestinal intolerance, which can be reduced by adopting the dose titration approach. If a subject develops an intolerable gastrointestinal adverse reaction during the dose titration phase, at the investigator's discretion, the dose may be decreased to the previous tolerated dose and administered for 2 weeks, then increased again in accordance with the protocol, and the treatment will be completed in accordance with the original protocol. If the subject is still intolerant, down-titrate to the previous highest tolerated dose to maintain treatment until completion of the study.

Treatment of missed dose

In case of a missed dose, it should be made up as soon as possible if there are at least 3 days (72 h) before the next scheduled dose. If there are less than 3 days (72 h) before the next scheduled dose, no make-ups should be made and the next scheduled dose is regularly administered. One missed dose of medication should not affect the subsequent scheduled dosing dates.

Rescue therapy

T2DM subjects with fasting glucose meeting the following criteria can be treated concomitantly with hypoglycemic agent, which is gradually increased to the maximum prescribed dose (metformin [preferred]); and under special circumstances, the investigator can decide on whether to perform salvage therapy based on the subject's condition and routine clinical practice.

- Weeks 1 to 12: FPG >13.9 mmol/L (250 mg/dL) (determined by two FPG measurements on different days)
- Weeks 13 to 57: FPG >11.1 mmol/L (200 mg/dL) (determined by two FPG measurements on different days)

During the course of the study, subjects should undergo FPG retesting if SMBG FPG is found to exceed the above values, or if the investigator finds that FPG exceeds the above values with no apparent causative factors. The status of medication compliance and changes in diet and exercise shall also be checked and adjusted accordingly. FPG retesting is performed after at least 3 days of adjustment to determine whether salvage therapy for hyperglycemia should be performed.

Investigators should document the use of salvage therapies in the original medical record in detail, including the reason for the medication, the dose of the medication, the duration of the medication, and the frequency of the medication. Those subjects who receive salvage therapy during the double-blind core treatment period will be discontinued from study drug upon completion of the double-blind core treatment, and the study is completed after a 5-week safety follow-up period after the last dose. Subjects treated with salvage therapy during the open-label treatment period should continue to attend the visits as specified in the protocol until completion of the clinical study.

Complete eligibility criteria

Subjects must meet all of the following inclusion criteria to be included in this study:

1) Body mass index (BMI) ≥ 20.0 kg/m^2 and ≤ 35.0 kg/m^2;
2) Meet the Diagnostic Criteria for Diabetes Mellitus promulgated by the World Health Organization (WHO) in 1999, have been diagnosed with T2DM for at least 3 months and received nutrition and exercise interventions for 8 weeks prior to screening, and meet one of the following criteria:
3) No use of any hypoglycemic agents in the previous 12 weeks, or
4) Use of only one oral hypoglycemic agent within the previous 12 weeks, but no further use within 2 weeks prior to screening;
5) HbA1c ≥ 7.5% and ≤ 11.0% at screening (local laboratory) and HbA1c ≥ 7.0% and ≤ 10.5% before randomization (central laboratory);
6) Fasting plasma glucose (FPG) ≤ 13.9 mmol/L at screening and pre-randomization (local laboratory);
7) Willing and able to perform self-monitoring of blood glucose (SMBG) and record diary cards on time;
8) Fully understand the study objectives, able to communicate well with the investigator, and able to understand and comply with the requirements set forth for this study.

Subjects must be excluded from the study if they meet *any* of the following criteria:

1) Prior diagnosis of type 1 diabetes mellitus or other types of diabetes mellitus;
2) Use of GLP-1 analogs or DPP-4 inhibitors within 3 months prior to screening;
3) Use of any insulin preparation within 6 months prior to screening (except for short-term insulin therapy related to complications, i.e., total treatment duration ≤ 14 days);
4) History of diabetic ketoacidosis (DKA), hyperosmolar hyperglycemic state (HHS) and diabetic lactic acidosis (DLA) within 6 months prior to screening;
5) Presence of severe chronic complications of diabetes mellitus (e.g., proliferative retinopathy or macular degeneration, severe diabetic neuropathy, diabetic foot, etc.) within 6 months prior to screening;
6) Confirmed or suspected grade 3 hypoglycemia episodes within 6 months prior to screening, or 2 or more hypoglycemia episodes (blood glucose < 3.9 mmol/L) within 1 month prior to screening;
7) History of severe trauma or serious infection or surgery that may affect glycemic control within 1 month prior to screening;
8) History of uncontrolled hyperthyroidism or hypothyroidism, except those subjects who have received stable dose of thyroid replacement therapy for at least 3 months prior to screening and whose thyroid function test results (thyroid-stimulating hormone [TSH]/free tri-iodothyronine [FT3]/free tetra-iodothyronine [FT4]) are in the normal range;
9) Prior personal or family history of medullary thyroid carcinoma (MTC) or multiple endocrine neoplasia syndrome type 2 (MEN2);
10) Medical or non-medical weight management, including unapproved weight management medications or products, within 3 months prior to screening, or a change in body weight of more than 5% (as self-reported by the subject) within 3 months prior to screening;
11) Prior history of significant cardiovascular disease, defined as:

a) History of myocardial infarction, coronary angioplasty or bypass grafting, heart valve disease or heart valve repair surgery, clinically significant unstable arrhythmia, unstable angina, transient ischemic attack, or cerebral vascular accident within 6 months prior to screening;
b) Congestive heart failure with a New York Heart Association (NYHA) classification of class III or IV (see Appendix VII);

12) Comorbid poorly controlled respiratory diseases, including but not limited to chronic obstructive pulmonary disease, bronchial asthma, and active tuberculosis, within 6 months prior to screening;
13) History of severe gastrointestinal disease (e.g., active ulcers) or gastrointestinal surgery (other than appendectomy or cholecystectomy) or clinically significant gastric emptying abnormalities (e.g., pyloric obstruction, gastroparesis) or chronic use of medications that have a direct effect on gastrointestinal motility within 6 months prior to screening;
14) Prior history of acute and chronic pancreatitis, symptomatic gallbladder disease, pancreatic injury and other factors that may contribute to a high risk of pancreatitis;
15) Prior diagnosis of malignant tumor (except cured basal cell carcinoma of the skin or carcinoma in situ of the cervix) within 5 years;
16) Pre-existing uncontrolled psychiatric and neurological disorders;
17) Positive hepatitis B surface antigen [HBsAg] (except for those with quantitative hepatitis B virus [HBV] deoxyribonucleic acid [DNA] test results below the lower limit of the reference range of the assay), or positive hepatitis C virus [HCV] antibody, or positive human immunodeficiency virus [HIV] antibody at the time of screening;
18) Screening or pre-randomization test results meeting any of the following criteria:
    a) Uncontrolled hypertension: systolic blood pressure ≥160 mmHg and/or diastolic blood pressure ≥ 100 mmHg;
    b) Liver disease or impaired liver function, such as alanine aminotransferase (ALT) or aspartate aminotransferase (AST) ≥ 2.5 times the upper limit of normal (ULN);
    c) Impaired pancreatic function, with blood amylase or blood lipase ≥ 1.5 times the ULN;
    d) Impaired renal function, i.e., estimated glomerular filtration rate (eGFR) ≤ 45 mL/min/1.73 m^2 (Modification of Diet in Renal Disease [MDRD] equation);
    e) Dyslipidemia, such as fasting triglycerides (TG) > 5.65 mmol/L;
    f) Fasting C-peptide < 0.8 ng/mL (or 0.26 nmol/L);
    g) Total bilirubin (TBIL) ≥ 3 times the ULN;
    h) Hemoglobin (Hb) <110 g/L (male) or <100 g/L (female);
    i) Calcitonin ≥ 50 ng/L;
19) Presence of significant hematologic disorders (e.g., aregenerative anaemia, myelodysplastic syndromes, etc.), or any disease causing hemolysis or red blood cell instability, or hemoglobinopathies that may affect the measurement of the HbA1c level (e.g., thalassemia, sickle cell anemia, sideroblastic anemia, etc.);
20) Prior history of alcohol or drug abuse (consumption of more than 14 units of alcohol per week [1 unit = 360 mL of beer containing 5% alcohol, 45 mL of spirits containing 40% alcohol, or 150 mL of wine containing 12% alcohol]);
21) Prolonged (≥ 7 consecutive or cumulative days) administration of systemic corticosteroids or growth hormones within 2 months prior to screening (with mode of administration including, but not limited to, intravenous administration, oral administration);
22) Subjects with known or suspected hypersensitivity to GLP-1 analogs or excipients;
23) Use of any investigational drug, vaccine or medical device within 3 months prior to screening;
24) Blood donation or significant blood loss (>400 mL), receipt of blood transfusion or use of blood products within 3 months prior to screening;
25) Female subjects being pregnant or breastfeeding at the time of screening, or subjects (including female partners of male subjects) who have plans for childbearing or sperm and egg donation from within 1 month prior to informed consent to 3 months after the last dose, or subjects of childbearing potential who refuse to use at least one effective contraceptive drug or device;
26) Possibility of failure to complete this study for other reasons, or unsuitable for the study in the opinion of the investigator.

Hypothesis testing and multiplicity

This is a pivotal study to evaluate the efficacy and safety of Ecnoglutide Injection, with the primary efficacy endpoint of changes in HbA_{1c} after 24 weeks of treatment from the baseline. The statistical hypotheses are as follows:

- null hypothesis H_0: $\mu_T - \mu_C \geq \delta$ (-0.5%)
- alternative hypothesis H_1: $\mu_T - \mu_C < \delta$ (-0.5%)

Where μ_T is the overall average change in HbA_{1c} after 24 weeks of treatment with 1.2 mg or 0.6 mg from baseline in the Ecnoglutide study group, μ_C is the overall average change in average HbA_{1c} after 24 weeks of administration from baseline in the combined placebo group, and δ is the superiority margin, which is taken as -0.5% in this study.

This study contains Ecnoglutide 1.2 mg and Ecnoglutide 0.6 mg study groups. Hypothesis testing will be performed on the two study groups separately based on the estimates, controlling for overall type I error of no more than 0.025 (one-sided) by the sequential test method, i.e., the difference between the 1.2 mg study group and the placebo group will be tested first, and if the H_0 is rejected, the test will continue to be performed between the 0.6 mg study group and the placebo group, otherwise the test will be stopped. The nominal significance level of the each test is 0.025 (one-sided), and H_0 could be rejected if the test $p<0.025$ (one-sided) or the upper limit of the two-sided 95% confidence interval for the inter-group difference $(\mu_T-\mu_C) <\delta$.

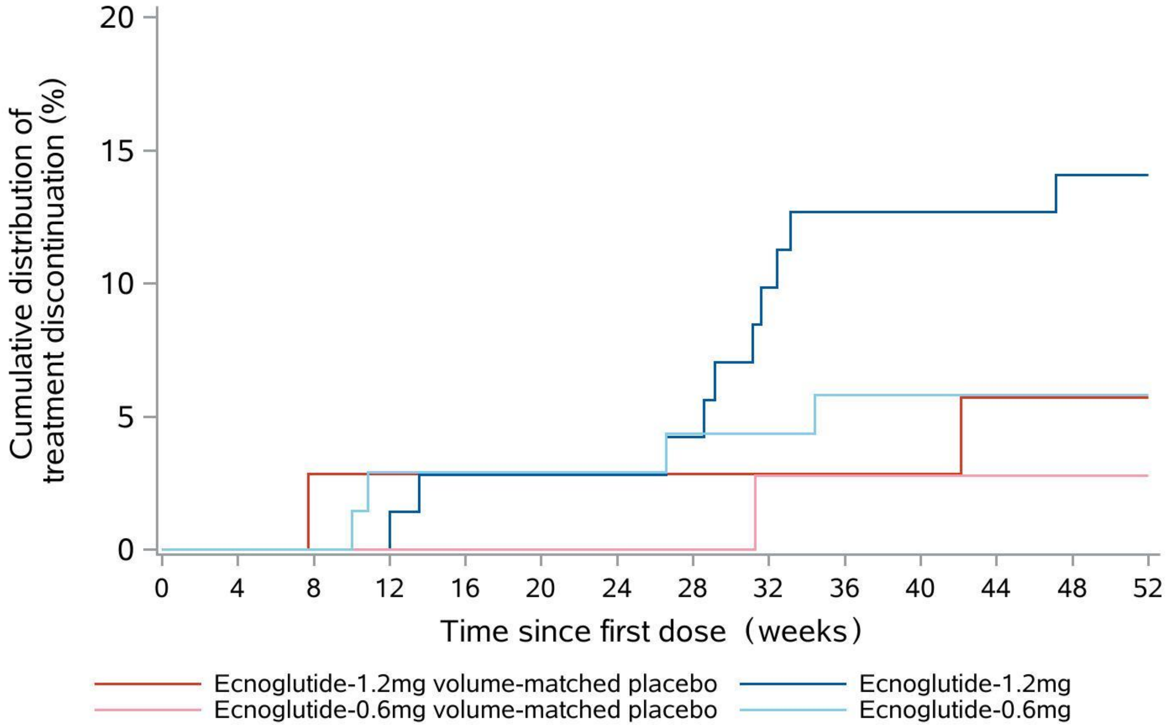

Supplementary Fig. 1: Time from treatment initiation to discontinuation

The cumulative distribution of treatment discontinuation in each group was estimated by the Kaplan-Meier method. Source data are provided in the Source Data file.

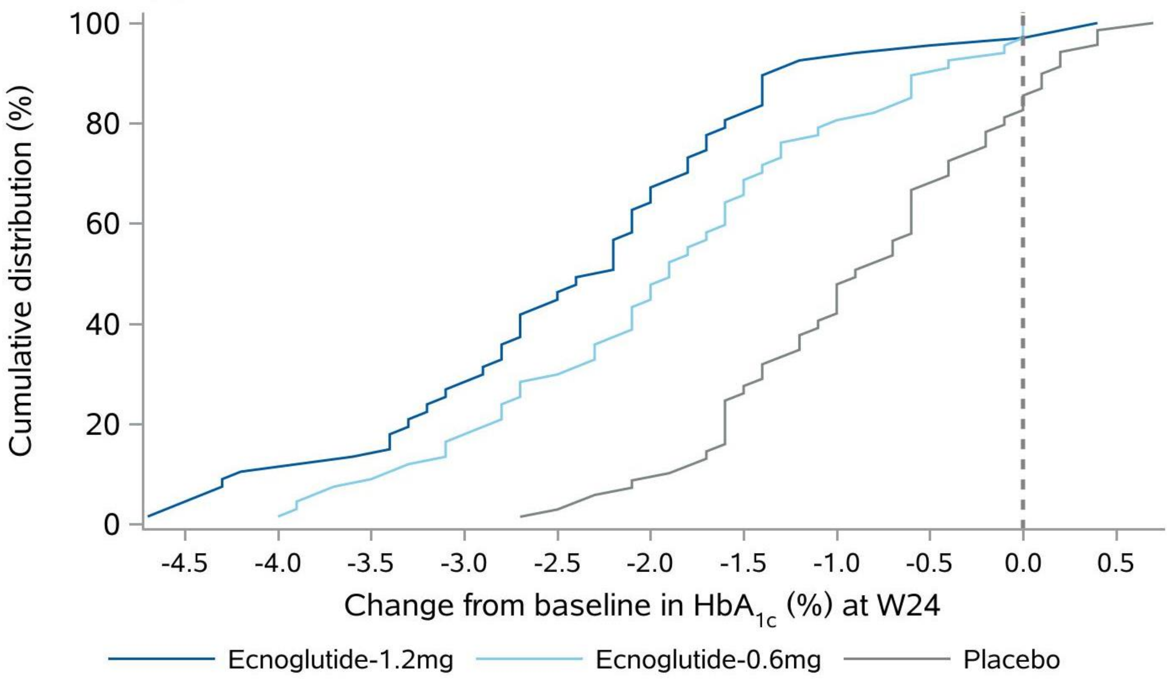

Supplementary Fig. 2: Cumulative distribution of change from baseline in HbA$_{1c}$ at week 24

Source data are provided as the Source Data file.

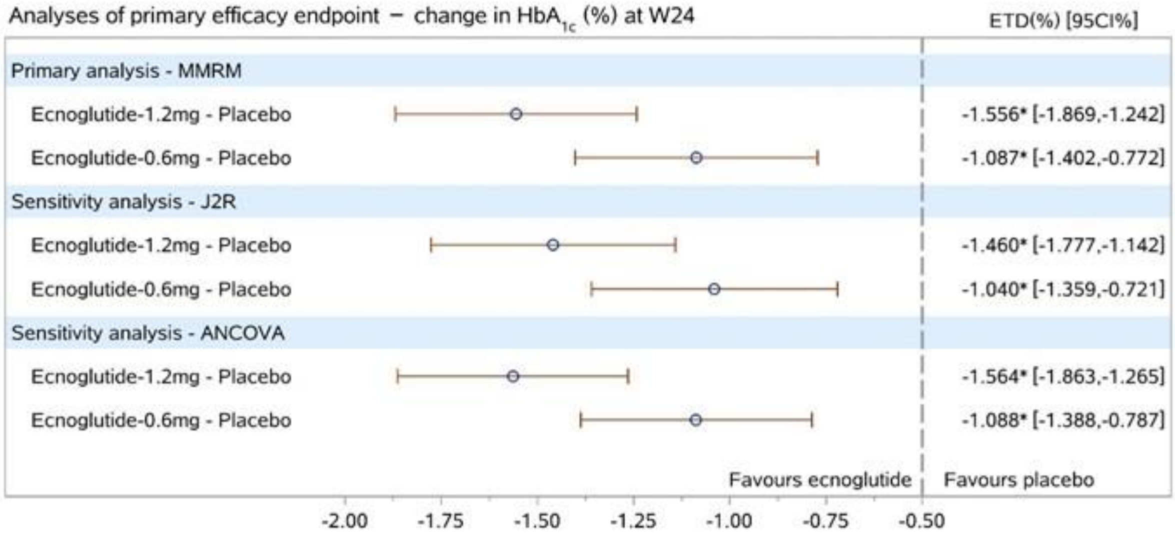

Supplementary Fig. 3: Sensitivity analysis of estimated treatment differences for change from baseline in HbA_{1c} at week 24

Missing data in primary analysis – MMRM and Sensitivity analysis – ANCOVA were imputed using multiple imputation methods under the assumption of missing at random (MAR). Missing data in ecnoglutide groups after intercurrent events in Sensitivity analysis – J2R were imputed based on the placebo group. ANCOVA=analysis of covariance. ETD=estimated treatment difference. HbA_{1c}=glycated haemoglobin. J2R=jump to reference. MMRM=Mixed model for repeated measures. *P<0.025.

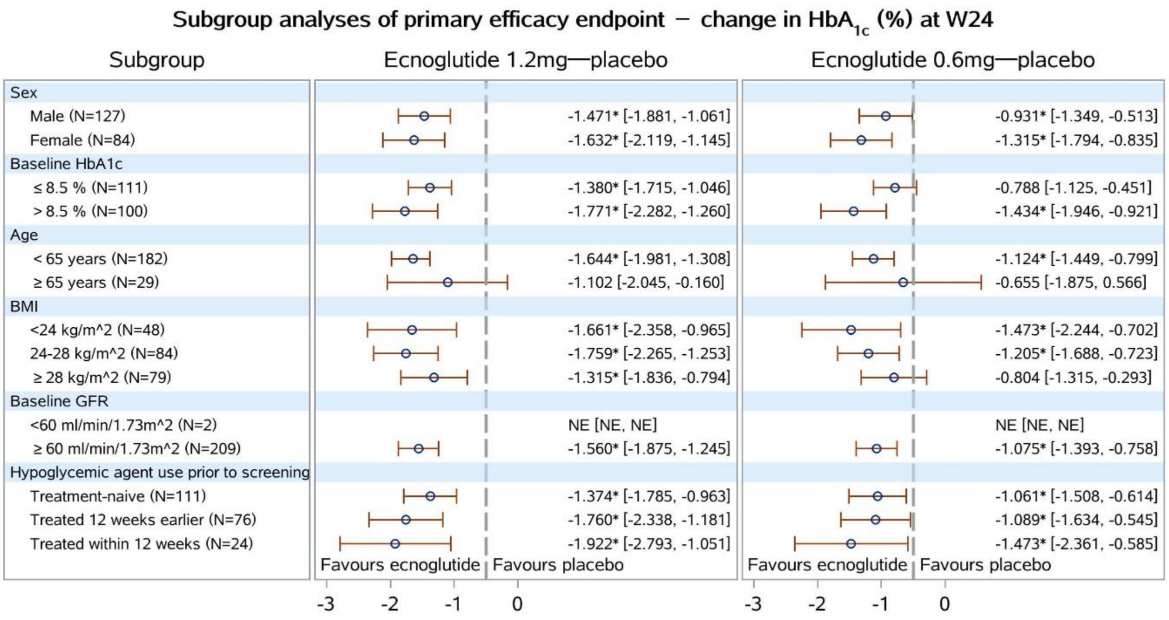

Supplementary Fig. 4: Subgroup analysis of estimated treatment difference for change from baseline in HbA_{1c} at week 24

Baseline was defined as the most recent non-missing measurement before randomisation. Imputation and analysis methods were the same as the primary analysis. The subgroup of ≥65 years could not be imputed by multiple imputation methods, and the calculation was based on non-missing data only. The subgroup analysis by hypoglycaemic agent use prior to screening was conducted *post-hoc*.

BMI=body-mass index. GFR= glomerular filtration rate. HbA_{1c}=glycated haemoglobin. NE=Not Estimable.

*P<0.025

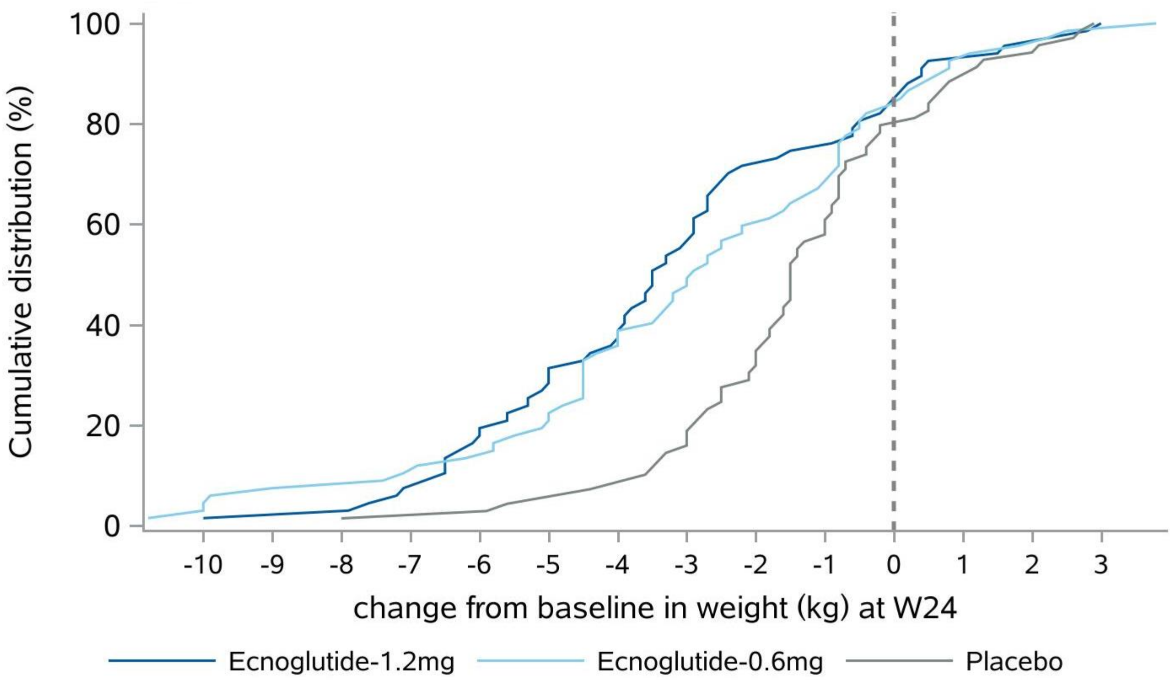

Supplementary Fig. 5: Cumulative distribution of change from baseline in bodyweight at week 24

Source data are provided as the Source Data file.

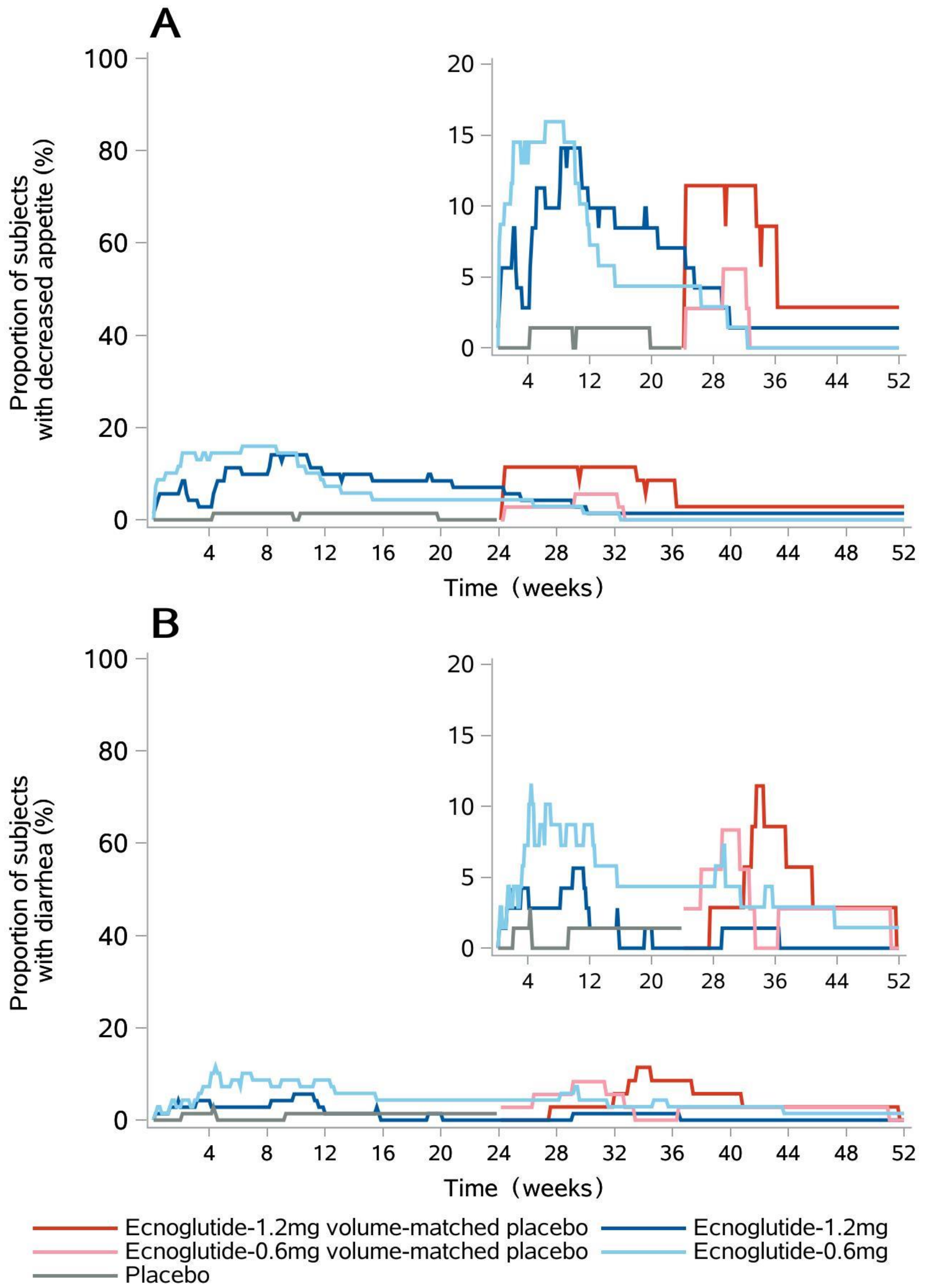

Supplementary Fig. 6: Incidences of decreased appetite and diarrhoea over time

(A) Incidence of decreased appetite over time in each group. (B) Incidence of diarrhoea over time in each group. To improve clarity, a small-scale figure is included in the top-right corner of each panel. Source data are provided as the Source Data file.

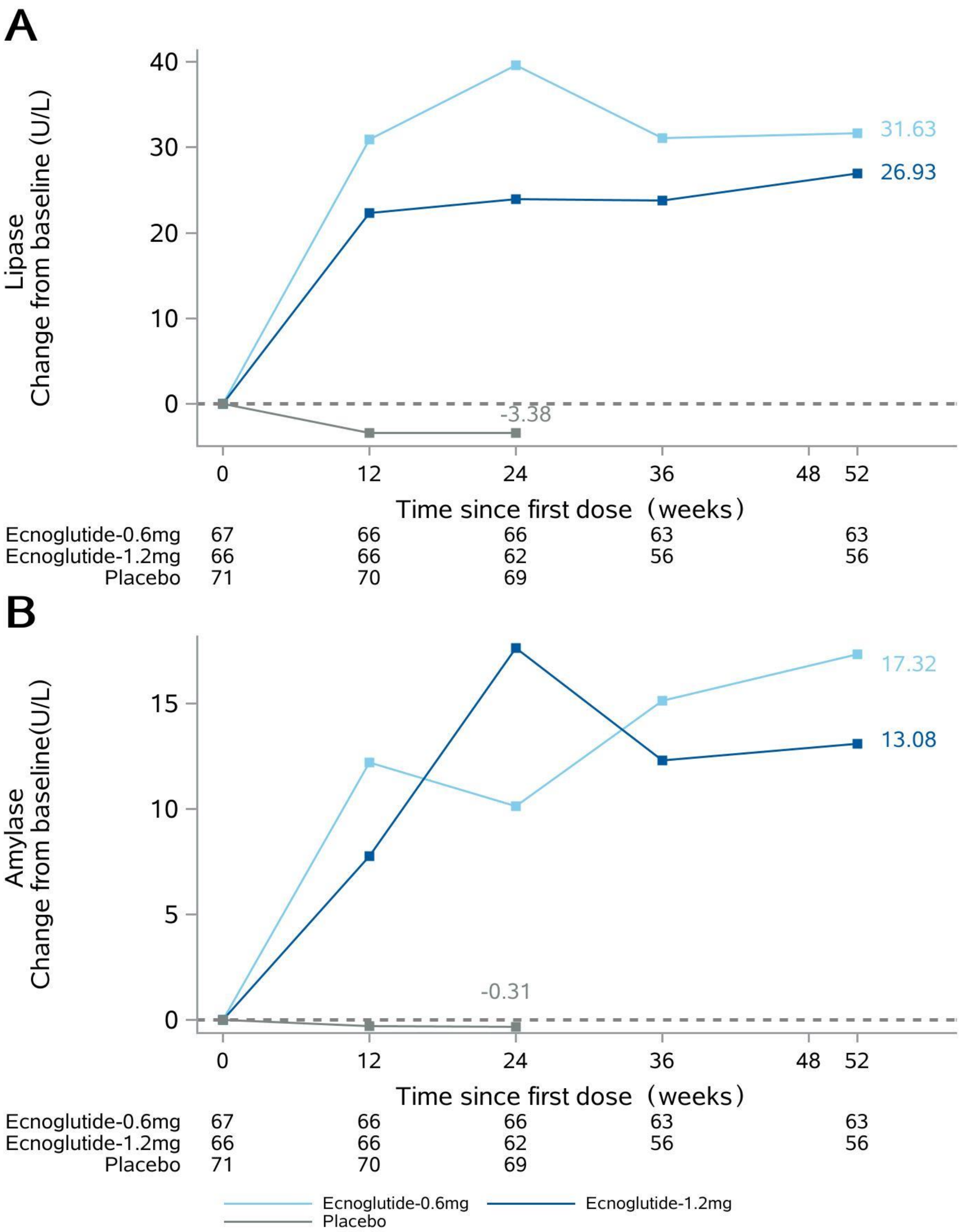

Supplementary Fig. 7: Changes from baseline in lipase and amylase over time

(A) Change from baseline in lipase over time in each group. (B) Change from baseline in amylase over time in each group. Source data are provided as the Source Data file.

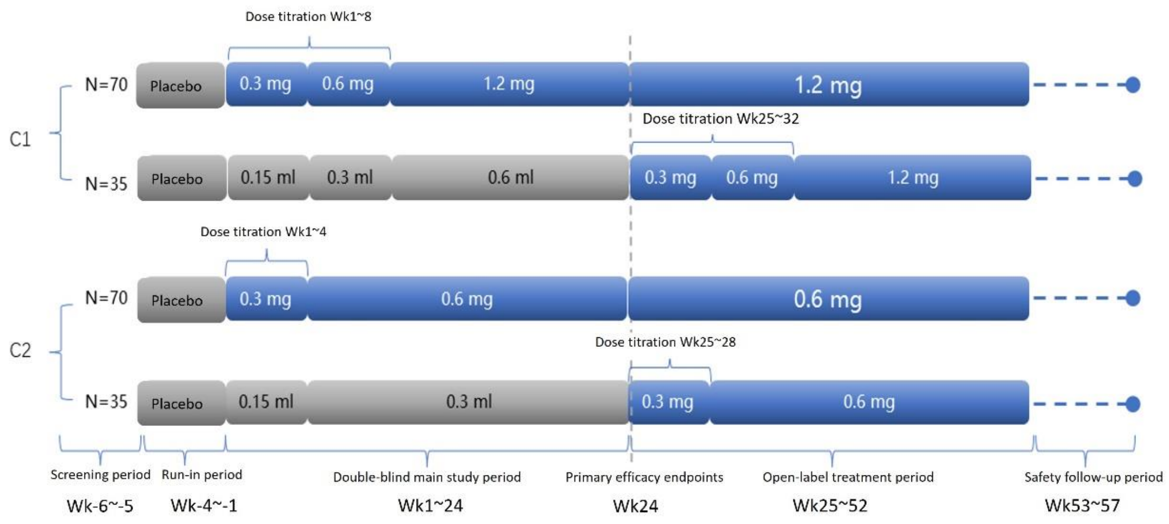

Supplementary Fig. 8: Study design

Supplementary Table 1: Additional efficacy measures at week 24

	Ecnoglutide 1.2 mg (N=71)			Ecnoglutide 0.6 mg (N=69)			Placebo (N=71)
	LSM change from baseline (95% CI) or n (%)	ETD or odds ratio (95% CI)	p value	LSM change from baseline (95% CI), or n (%)	ETD or odds ratio (95% CI)	p value	LSM change from baseline (95% CI) or n (%)
HbA$_{1c}$<7.0%	57 (80.3%)	17.96 (7.582 to 42.523)	<0.0001	47 (68.1%)	9.22 (4.128 to 20.574)	<0.0001	15 (21.1%)
HbA$_{1c}$≤6.5%	54 (76.1%)	24.04 (9.673 to 59.729)	<0.0001	36 (52.2%)	8.02 (3.391 to 18.950)	<0.0001	9 (12.7%)
HbA$_{1c}$<5.7%	25 (35.2%)	–	–	7 (10.1%)	–	–	0
The composite endpoint of HbA$_{1c}$<7.0%, no severe hypoglycaemia, and no bodyweight gain	51 (71.8%)	12.47 (5.505 to 28.267)	<0.0001	40 (58.0%)	6.64 (3.017 to 14.631)	<0.0001	13 (18.3%)
≥5% reduction in bodyweight	31 (43.7%)	6.20 (2.498 to 15.401)	<0.0001	27 (39.1%)	5.73 (2.267 to 14.503)	0.0002	8 (11.3%)
≥10% reduction in bodyweight	7 (9.9%)	6.93 (0.821 to 58.441)	0.0752	7 (10.1%)	7.64 (0.904 to 64.557)	0.0619	1 (1.4%)
Fasting insulin (pmol/L)	8.276 (-4.702 to 21.255)	7.484 (-10.728 to 25.696)	0.4187	3.949 (-9.095 to 16.993)	3.157 (-15.191 to 21.505)	0.7347	0.792 (-12.018 to 13.602)
HOMA-IR	-1.124 (-1.974 to -0.275)	-0.628 (-1.819 to 0.564)	0.3002	-1.291 (-2.137 to -0.444)	-0.794 (-1.988 to 0.4)	0.1912	-0.497 (-1.334 to 0.341)
HOMA-β	65.466 (45.152 to 85.78)	51.849 (23.333 to 80.366)	0.0004	57.445 (37.176 to 77.714)	43.829 (15.193 to 72.464)	0.0029	13.617 (-6.445 to 33.678)
LDL-C (mmol/L)	-0.346 (-0.492 to -0.201)	-0.182 (-0.386 to 0.022)	0.0796	-0.325 (-0.471 to -0.179)	-1.161 (-0.366 to 0.043)	0.1217	-0.164 (-0.307 to-0.021)
TG (mmol/L)	-0.248 (-0.478 to -0.018)	-0.492 (-0.815 to -0.169)	0.0030	0.096 (-0.134 to 0.326)	-0.148 (-0.471 to 0.175)	0.3672	0.244 (0.017 to 0.470)
HDL-C (mmol/L)	-0.020 (-0.066 to 0.026)	0.006 (-0.059 to 0.071)	0.8617	-0.030 (-0.076 to 0.016)	-0.005 (-0.069 to 0.060)	0.8903	-0.026 (-0.071 to -0.020)

Note: For each continuous parameter, analysis of covariance (ANCOVA) was used, which regarded its change from baseline at week 24 as the dependent variable, and its baseline value and treatment group as the independent variables. P-values for continuous endpoints were obtained by two-sided t-tests based on ANCOVA and p-values for the discrete endpoints were obtained by two-sided z-tests based on logistic regressions, without multiplicity adjusted for all endpoints listed here.
ANCOVA=analysis of covariance. CI=confidence interval. HbA$_{1c}$=haemoglobin A1C. HDL-C=high-density lipoprotein cholesterol. HOMA-β=homeostasis model assessment of β-cell function. HOMA-IR=homeostasis model assessment of insulin resistance. LDL-C=low-density lipoprotein cholesterol. LSM=least square mean. TG=triglycerides.

Supplementary Table 2: Efficacy measures at week 52

	Placebo to ecnoglutide 1.2 mg (N=31)	Ecnoglutide 1.2 mg (N=64)	Placebo to ecnoglutide 0.6 mg (N=34)	Ecnoglutide 0.6 mg (N=66)
HbA_{1c} (%)	-2.45 (0.947)	-2.34 (1.120)	-2.05 (0.849)	-2.01 (0.934)
HbA_{1c}<7.0%	26 (83.9%)	52 (81.3%)	26 (76.5%)	51 (77.3%)
HbA_{1c}≤6.5%	23 (74.2%)	49 (76.6%)	17 (50.0%)	37 (56.1%)
HbA_{1c}<5.7%	15 (48.4%)	14 (21.9%)	4 (11.8%)	8 (12.1%)
Fasting plasma glucose (mmol/L)	-3.076 (1.915)	-3.226 (1.939)	-3.681 (1.657)	-3.168 (2.035)
2h-postprandial blood glucose (mmol/L)	-7.509 (4.020)	-6.656 (3.974)	-7.494 (3.588)	-6.505 (3.876)
7-Point SMBG (mmol/L)				
Mean glucose	-4.451 (2.523)	-4.060 (2.369)	-3.839 (2.460)	-3.929 (3.266)
Postprandial glucose excursion	-1.813 (1.709)	-1.489 (1.884)	-1.043 (1.631)	-1.166 (2.359)
Bodyweight (kg)	-4.62 (3.555)	-3.84 (3.281)	-2.65 (3.280)	-3.82 (3.754)
Waist circumference (cm)	-3.85 (3.888)	-4.45 (4.092)	-2.49 (3.054)	-3.90 (4.075)
Hip circumference (cm)	-3.15 (2.205)	-2.89 (3.166)	-1.84 (2.558)	-2.84 (3.269)
Fasting insulin (pmol/L)	-7.847 (37.558)	0.212 (53.276)	-5.566 (19.053)	-15.018 (90.533)
HOMA-IR	-1.993 (2.781)	-1.627 (3.879)	-2.087 (2.269)	-2.589 (5.657)
HOMA-β	66.902 (91.735)	62.017 (82.281)	52.161 (104.353)	44.354 (96.868)
LDL-C (mmol/L)	-0.343 (0.775)	-0.244 (0.757)	-0.355 (0.725)	-0.315 (0.709)
TG (mmol/L)	-0.605 (1.145)	-0.465 (1.059)	-0.119 (0.639)	-0.262 (0.821)
HDL-C (mmol/L)	0.026 (0.193)	0.020 (0.198)	-0.041 (0.211)	0.001 (0.193)

Notes: Data are mean (SD) change from baseline or n (%), without imputation for missing data. HbA_{1c}=glycated haemoglobin. HDL-C=high-density lipoprotein cholesterol. HOMA-β=homeostasis model assessment of β-cell function. HOMA-IR=homeostasis model assessment of insulin resistance. LDL-C=low-density lipoprotein cholesterol. LSM=least square mean. SMBG=self-measuring of blood glucose. SD=standard deviation. TG=triglycerides.

Supplementary Table 3: Pulse rate and blood pressure at baseline and week 24

	Ecnoglutide 1.2 mg (N=71)	Ecnoglutide 0.6 mg (N=69)	Placebo (N=71)
Pulse rate, mean (SD), beats/min			
Baseline	76.3 (9.48)	79.8 (10.84)	78.2 (9.76)
Week 24	81.6 (8.55)	82.8 (9.15)	76.5 (10.55)
Change from baseline	5.4 (9.07)	3.0 (9.84)	-1.6 (9.20)
Systolic blood pressure, mean (SD), mmHg			
Baseline	124.1 (12.81)	124.0 (12.95)	122.0 (13.30)
Week 24	119.0 (12.80)	119.8 (13.04)	119.3 (12.64)
Change from baseline	-5.2 (12.00)	-4.4 (13.37)	-2.1 (13.40)
Diastolic blood pressure, mean (SD), mmHg			
Baseline	80.7 (8.90)	82.6 (8.32)	81.5 (8.31)
Week 24	79.5 (8.18)	80.2 (8.57)	78.4 (8.65)
Change from baseline	-1.1 (7.58)	-2.5 (6.89)	-2.9 (8.13)

SD=standard deviation.

Supplementary Table 4: Treatment-emergent adverse events (TEAEs) during the open-label treatment period

Participants, n (%)	Placebo to ecnoglutide 1.2 mg (N=31)	Ecnoglutide 1.2 mg (N=64)	Placebo to ecnoglutide 0.6 mg (N=34)	Ecnoglutide 0.6 mg (N=66)
Any TEAEs	27 (87.1)	46 (71.9)	29 (85.3)	43 (65.2)
Treatment-related	18 (58.1)	15 (23.4)	18 (52.9)	22 (33.3)
Grade ≥3 TEAEs	3 (9.7)	4 (6.3)	3 (8.8)	7 (10.6)
Treatment-related	2 (6.5)	1 (1.6)	1 (2.9)	2 (3.0)
Serious TEAEs	3 (9.7)	3 (4.7)	2 (5.9)	5 (7.6)
Treatment-related	2 (6.5)	0	0	0
TEAEs leading to treatment discontinuation	0	1 (1.6)	0	1 (1.5)
TEAEs leading to death	0	0	0	0
TEAEs of special interests occurring in ≥5% of participants in any treatment group (preferred term)				
Decreased appetite	6 (19.4)	1 (1.6)	2 (5.9)	0
Diarrhoea	5 (16.1)	1 (1.6)	5 (14.7)	5 (7.6)
Hypoglycaemia (blood glucose <3.9 mmol/L)	1 (3.2)	4 (6.3)	2 (5.9)	2 (3.0)
Nausea	1 (3.2)	1 (1.6)	4 (11.8)	1 (1.5)

Supplementary Table 5: Treatment-emergent adverse events (TEAEs) during the entire treatment period

Participants, n (%)	Placebo to ecnoglutide 1.2 mg (N=35)	Ecnoglutide 1.2 mg (N=71)	Placebo to ecnoglutide 0.6 mg (N=36)	Ecnoglutide 0.6 mg (N=69)
Any TEAEs	33 (94.3)	67 (94.4)	33 (91.7)	60 (87.0)
Treatment-related	21 (60.0)	47 (66.2)	23 (63.9)	47 (68.1)
Grade ≥3 TEAEs	4 (11.4)	9 (12.7)	6 (16.7)	9 (13.0)
Treatment-related	2 (5.7)	4 (5.6)	1 (2.8)	2 (2.9)
Serious TEAEs	3 (8.6)	6 (8.5)	6 (16.7)	7 (10.1)
Treatment-related	2 (5.7)	1 (1.4)	0	0
TEAEs leading to treatment discontinuation	0	2 (2.8)	1 (2.8)	2 (2.9)
TEAEs leading to death	0	0	0	0
TEAEs of special interests occurring in ≥5% of participants in any treatment group (preferred term)				
Decreased appetite	8 (22.9)	19 (26.8)	3 (8.3)	15 (21.7)
Diarrhoea	7 (20.0)	9 (12.7)	6 (16.7)	20 (29.0)
Nausea	4 (11.4)	10 (14.1)	5 (13.9)	6 (8.7)
Flatulence	3 (8.6)	1 (1.4)	1 (2.8)	0
Sinus bradycardia	3 (8.6)	0	0	1 (1.4)
Gastroesophageal reflux disease	1 (2.9)	4 (5.6)	1 (2.8)	4 (5.8)
Hypoglycaemia (blood glucose <3.9 mmol/L)	1 (2.9)	5 (7.0)	3 (8.3)	3 (4.3)
Vomiting	1 (2.9)	4 (5.6)	0	4 (5.8)
Abdominal distension	0	6 (8.5)	1 (2.8)	3 (4.3)
Abdominal pain	0	4 (5.6)	0	1 (1.4)

Supplementary Note 1: CONSORT Reporting Checklist

	Item Description	Location (or reason for not reporting)
**Title and Abstract**		
1a. Title	Identification as a randomised trial.	Page 1
1b. Structured Abstract	Structured summary of the trial design, methods, results, and conclusions.	Page 3, but the abstract is unstructured based on the journal requirements.
**Open Science**		
2. Trial Registration	Name of trial registry, identifying number (with URL) and date of registration.	Page 20
Protocol and statistical analysis plan	Where the trial protocol and statistical analysis plan can be accessed.	Provided during submission
4. Data sharing	Where and how the individual de-identified participant data (including data dictionary), statistical code and any other materials can be accessed.	Page 20
5. Funding and Conflicts of Interest		
5a. Funding	Sources of funding and other support (eg, supply of drugs), and role of funders in the design, conduct, analysis, and reporting of the trial.	Pages 24 to 25
5b. Conflicts of interest	Financial and other conflicts of interest of the manuscript authors.	Page 25
**Introduction**		
6. Background and rationale	Scientific background and rationale.	Pages 4 to 5
7. Objectives	Specific objectives related to benefits and harms.	Page 5
**Methods**		
8. Patient and public involvement	Details of patient or public involvement in the design, conduct and reporting of the trial.	N/A
9. Trial Design	Description of trial design including type of trial (eg, parallel group, crossover), allocation ratio, and framework (eg, superiority, equivalence, non-inferiority, exploratory).	Pages 17 to 18
10. Changes to trial protocol	Important changes to the trial after it commenced including any outcomes or analyses that were not pre-specified, with reason.	N/A
11. Trial Setting	Settings (eg, community, hospital) and locations (eg, countries, sites) where the trial was conducted.	Page 17
12. Eligibility Criteria		
12a. Participants	Eligibility criteria for participants.	Page 18
12b. Other	If applicable, eligibility criteria for sites and for individuals delivering the interventions (eg, surgeons, physiotherapists).	N/A
13. Intervention and comparator	Intervention and comparator with sufficient details to allow replication. If relevant, where additional materials describing the intervention and comparator (eg, intervention manual) can be accessed.	Page 17
14. Outcomes	Prespecified primary and secondary outcomes, including the specific measurement variable (eg, systolic blood pressure), analysis metric (eg, change from baseline, final value, time to event), method of aggregation (eg, median, proportion), and time point for each outcome.	Page 18

15. Harms	How harms were defined and assessed (eg, systematically, non-systematically).	Pages 17 to 18
16. Sample Size		
16a. How sample size was determined	How sample size was determined, including all assumptions supporting the sample size calculation.	Page 19
16b. Interim analyses and stopping criteria	Explanation of any interim analyses and stopping guidelines.	N/A
17. Randomisation		
17a. Sequence Generation	Who generated the random allocation sequence and the method used.	Page 17
17b. Type of Randomisation	Type of randomisation and details of any restriction (eg, stratification, blocking, and block size).	Page 17
18. Allocation concealment mechanism	Mechanism used to implement the random allocation sequence (eg, central computer/telephone; sequentially numbered, opaque, sealed containers), describing any steps to conceal the sequence until interventions were assigned.	Page 17
19. Implementation	Whether the personnel who enrolled and those who assigned participants to the interventions had access to the random allocation sequence.	Page 17
20. Blinding		
20a. Who was blinded	Who was blinded after assignment to interventions (eg, participants, care providers, outcome assessors, data analysts).	Page 17
20b. How blinding was achieved	If blinded, how blinding was achieved and description of the similarity of interventions.	Page 17
21. Statistical methods		
21a. Comparing groups	Statistical methods used to compare groups for primary and secondary outcomes, including harms.	Pages 19 to 20
21b. Definition of who is included in each analysis	Definition of who is included in each analysis (e.g., all randomised participants), and in which group.	Page 20
21c. Missing Data	How missing data were handled in the analysis.	Pages 19 to 20
21d. Additional Analyses	Methods for any additional analyses (eg, subgroup and sensitivity analyses), distinguishing pre-specified from post hoc.	Page 20
22. Participant flow, including flow diagram		
22a. Participant Numbers	For each group, the numbers of participants who were randomly assigned, received intended intervention, and were analysed for the primary outcome.	Pages 5 to 6
22b. Losses and exclusions	For each group, losses and exclusions after randomisation, together with reasons.	Pages 5 to 6
23. Recruitment		
23a. Dates	Dates defining the periods of recruitment and follow-up for outcomes of benefits and harms.	Page 5
23b. Reasons for stopping recruitment	If relevant, why the trial ended or was stopped.	N/A
24. Intervention and comparator delivery		
24a. As Administered	Intervention and comparator as they were actually administered (eg, where appropriate, who delivered the	Page 5

	intervention/comparator, whether participants adhered, whether they were delivered as intended (fidelity)).	
24b. Concomitant Care	Concomitant care received during the trial for each group.	Page 6
25. Baseline Data	A table showing baseline demographic and clinical characteristics for each group.	Pages 6 to 7
26. Numbers analysed, outcomes, and estimation	For each primary and secondary outcome, by group:<br>• the number of participants included in the analysis.<br>• the number of participants with available data at the outcome time point.<br>• result for each group, and the estimated effect size and its precision (such as 95% confidence interval).<br>• for binary outcomes, presentation of both absolute and relative effect size.	Pages 7 to 14
27. Harms	All harms or unintended events in each group.	Pages 12 to 14
28. Ancillary Analyses	Any other analyses performed, including subgroup and sensitivity analyses, distinguishing pre-specified from post hoc.	Page 10
**Discussion**		
29. Interpretation	Interpretation consistent with results, balancing benefits and harms, and considering other relevant evidence.	Pages 14 to 16
30. Limitations	Trial limitations, addressing sources of potential bias, imprecision, generalisability, and, if relevant, multiplicity of analyses.	Page 16

Note: Page numbers within the checklist referred to the page numbers within the originally submitted manuscript.
