# Supplementary material for: Efficacy and safety of cAMP signalling-biased GLP-1 analogue ecnoglutide monotherapy versus placebo in patients with type 2 diabetes (EECOH-1): a multi-centre, randomised, double-blind, placebo-controlled, phase 3 trial
Source: Nat Commun. 2026 Jan 7;17:1420. doi: 10.1038/s41467-025-68165-7 (PMC12881390; doi:10.1038/s41467-025-68165-7)
Supplement: Supplementary file 2 — Reporting Summary [file 41467_2025_68165_MOESM2_ESM.pdf]

nature portfolio

Corresponding author(s): Dalong Zhu, Shaohui Bing

Last updated by author(s): Nov 14, 2025

Reporting Summary

Nature Portfolio wishes to improve the reproducibility of the work that we publish. This form provides structure for consistency and transparency in reporting. For further information on Nature Portfolio policies, see our Editorial Policies and the Editorial Policy Checklist.

Statistics

For all statistical analyses, confirm that the following items are present in the figure legend, table legend, main text, or Methods section.

n/a	Confirmed	
☐	☒	The exact sample size (*n*) for each experimental group/condition, given as a discrete number and unit of measurement
☐	☒	A statement on whether measurements were taken from distinct samples or whether the same sample was measured repeatedly
☐	☒	The statistical test(s) used AND whether they are one- or two-sided<br>*Only common tests should be described solely by name; describe more complex techniques in the Methods section.*
☐	☒	A description of all covariates tested
☐	☒	A description of any assumptions or corrections, such as tests of normality and adjustment for multiple comparisons
☐	☒	A full description of the statistical parameters including central tendency (e.g. means) or other basic estimates (e.g. regression coefficient) AND variation (e.g. standard deviation) or associated estimates of uncertainty (e.g. confidence intervals)
☐	☒	For null hypothesis testing, the test statistic (e.g. *F*, *t*, *r*) with confidence intervals, effect sizes, degrees of freedom and *P* value noted<br>*Give P values as exact values whenever suitable.*
☒	☐	For Bayesian analysis, information on the choice of priors and Markov chain Monte Carlo settings
☒	☐	For hierarchical and complex designs, identification of the appropriate level for tests and full reporting of outcomes
☒	☐	Estimates of effect sizes (e.g. Cohen's *d*, Pearson's *r*), indicating how they were calculated

Our web collection on statistics for biologists contains articles on many of the points above.

Software and code

Policy information about availability of computer code

Data collection	Date were collected using TrialOS Electronic Data Capture 5.0
Data analysis	Statistical analyses were completed using the statistical software SAS version 9.4 or above.

For manuscripts utilizing custom algorithms or software that are central to the research but not yet described in published literature, software must be made available to editors and reviewers. We strongly encourage code deposition in a community repository (e.g. GitHub). See the Nature Portfolio guidelines for submitting code & software for further information.

Data

Policy information about availability of data

All manuscripts must include a data availability statement. This statement should provide the following information, where applicable:

- Accession codes, unique identifiers, or web links for publicly available datasets
- A description of any restrictions on data availability
- For clinical datasets or third party data, please ensure that the statement adheres to our policy

The full dataset and protocol are not publicly available due to data privacy laws and contractual obligations. Sciwind Biosciences will provide de-identified individual participant data underlying the reported results upon request. Data are available after acceptance of this article with no expiration of data requests currently set. Requests should be made by contacting corresponding authors and will be evaluated within 6 months of receipt. Access will be provided after the proposed use of the data has been approved by a review committee and receipt of a signed data access agreement with Sciwind Biosciences.

Research involving human participants, their data, or biological material

Policy information about studies with human participants or human data. See also policy information about sex, gender (identity/presentation), and sexual orientation and race, ethnicity and racism.

Reporting on sex and gender	Male and female participants were enrolled in the study (56.5%, 60.6% and 63.4% male for ecnoglutide 0.6 mg, ecnoglutide 1.2 mg and placebo groups,respectively). Participants' gender was self-reported.
Reporting on race, ethnicity, or other socially relevant groupings	This study was conducted in China. 99.1% of participants were of Han ethnicity.
Population characteristics	Eligible male and female (non-pregnant and non-lactating) participants were 18-75 years of age (inclusive) and with a diagnosis of T2DM according to WHO criteria. In the three months prior to screening, they were to have been treated with diet and/or exercise alone or with one oral hypoglycemic agent.
Recruitment	The IRB-approved recruitment advertisement was distributed via flyers at clinics/hospitals/community centers, on public websites of clinics/hospitals/community centers, or digital news/social media. No potential self-selection bias is evident, although willingness to participate in a clinical trial and compliance with study procedures may not be homogenous. Investigators from 27 hospitals in China screened and enrolled participants into this study. Participants must meet the all the inclusion criteria and should not meet any one of the exclusion criteria as defined in the clinical study protocol.
Ethics oversight	The trial was conducted per the Declaration of Helsinki and International Conference on Harmonisation Guidelines for Good Clinical Practice. All participants provided written informed consent before study entry. The study protocol was approved by ethics committees at the following institutions: Nanjing Drum Tower Hospital, Nanjing First Hospital, Nanjing Jiangning Hospital, The Second Affiliated Hospital of Nanjing Medical University, The First Affiliated Hospital of Soochow University, Central Hospital Affiliated to Shandong First Medical University, Jining No.1 People's Hospital, Yiyang Central Hospital, The First Affiliated Hospital of Nanyang Medical College, Shiyan People's Hospital, The Second Hospital of Anhui Medical University, Binzhou Medical University Hospital, Shanghai Pudong New Area People's Hospital, Hebei Petro China Central Hospital, The First Affiliated Hospital of Henan University of Science and Technology, The Second Affiliated Hospital of Zhengzhou University, Yueyang People's Hospital, Yueyang Central Hospital, The Third Hospital of Changsha, Daqing People's Hospital, The People's Hospital of Liaoning Province, Yibin Second People's Hospital, The Affiliated Hospital of Xuzhou Medical University, Beijing Boai Hospital, Shijiazhuang People's Hospital, Nanyang Second General Hospital, and Pingxiang People's Hospital.

Note that full information on the approval of the study protocol must also be provided in the manuscript.

Field-specific reporting

Please select the one below that is the best fit for your research. If you are not sure, read the appropriate sections before making your selection.

☒ Life sciences ☐ Behavioural & social sciences ☐ Ecological, evolutionary & environmental sciences

For a reference copy of the document with all sections, see nature.com/documents/nr-reporting-summary-flat.pdf

Life sciences study design

All studies must disclose on these points even when the disclosure is negative.

Sample size	The sample size calculation assumed at least a -1·2% difference in mean change from baseline in HbA1c at week 24 between ecnoglutide groups and the pooled placebo group, a common standard deviation (SD) of 1·1%, and a drop-out rate of 20%. A sample size of 210 participants provided at least 90% power to establish superiority for an ecnoglutide dose compared with placebo (superiority margin of 0·5%) at a one-sided significance level of 0·025.
Data exclusions	Efficacy analyses were performed in the full analysis set (FAS), comprising all randomized participants who received ≥1 dose of study treatment. Safety analyses were conducted in the safety set, comprising all participants who received ≥1 dose of study treatment and safety evaluation after treatment initiation.
Replication	Results were consistent between the protocol-defined estimands.
Randomization	Participants were randomly assigned (2:2:1:1) to receive once-weekly subcutaneous injections of ecnoglutide (0·6 or 1·2 mg) or volume-matched placebo (0·6 or 1·2 mg), with stratification according to baseline HbA1c (≤8·5% or >8·5%), via an interactive web response system.
Blinding	This study was double-blinded. All investigators, participants, and the sponsor remained blinded to treatment assignment. Placebo and active drug were provided in injector pens, identical in appearance.

Reporting for specific materials, systems and methods

We require information from authors about some types of materials, experimental systems and methods used in many studies. Here, indicate whether each material, system or method listed is relevant to your study. If you are not sure if a list item applies to your research, read the appropriate section before selecting a response.

Materials & experimental systems

n/a	Involved in the study	
☒	☐	Antibodies
☒	☐	Eukaryotic cell lines
☒	☐	Palaeontology and archaeology
☒	☐	Animals and other organisms
☐	☒	Clinical data
☒	☐	Dual use research of concern
☒	☐	Plants

Methods

n/a	Involved in the study	
☒	☐	ChIP-seq
☒	☐	Flow cytometry
☒	☐	MRI-based neuroimaging

Clinical data

Policy information about clinical studies

All manuscripts should comply with the ICMJE guidelines for publication of clinical research and a completed CONSORT checklist must be included with all submissions.

Clinical trial registration	www.chinadrugtrials.org.cn, CTR20223156; clinicaltrials.gov, NCT05680155.
Study protocol	The study protocol is not publicly available and has been provided for peer review.
Data collection	Between 29 December 2022 and 12 June 2024, 211 adult subjects with T2DM were enrolled and data were collected in 27 China centers.
Outcomes	The primary efficacy endpoint was change from baseline in HbA1c at week 24, assessed by the central laboratory. The secondary efficacy endpoints included proportions of participants who achieved an HbA1c level of <7·0% and ≤6·5% at weeks 24 and 52; proportion of participants who achieved a composite endpoint of HbA1c <7·0%, no severe hypoglycaemia, and no bodyweight gain at week 24; changes from baseline at weeks 24 and 52 in FPG, 2-h postprandial plasma glucose (2h-PPG), seven-point self-monitored blood glucose (SMBG) profiles, fasting insulin, homeostasis model assessments of β-cell function (HOMA-β) and insulin resistance (HOMA-IR), blood lipids, bodyweight, waist circumference, and hip circumference. Safety endpoints included the incidences of treatment-emergent adverse events (TEAEs), serious TEAEs, and TEAEs of special interest (hypoglycaemia, cardiovascular events, gastrointestinal events, pancreatitis, and gallbladder-related disorders). Other safety measurements included vital signs, physical examinations, 12-lead electrocardiograms (ECG), and laboratory assessments.

Plants

Seed stocks	*Report on the source of all seed stocks or other plant material used. If applicable, state the seed stock centre and catalogue number. If plant specimens were collected from the field, describe the collection location, date and sampling procedures.*
Novel plant genotypes	*Describe the methods by which all novel plant genotypes were produced. This includes those generated by transgenic approaches, gene editing, chemical/radiation-based mutagenesis and hybridization. For transgenic lines, describe the transformation method, the number of independent lines analyzed and the generation upon which experiments were performed. For gene-edited lines, describe the editor used, the endogenous sequence targeted for editing, the targeting guide RNA sequence (if applicable) and how the editor was applied.*
Authentication	*Describe any authentication procedures for each seed stock used or novel genotype generated. Describe any experiments used to assess the effect of a mutation and, where applicable, how potential secondary effects (e.g. second site T-DNA insertions, mosiacism, off-target gene editing) were examined.*
